# Diagnostic and Therapeutic Challenges of Hereditary Tyrosinemia Type 1 in Lebanon: A 12-Year Retrospective Review

**DOI:** 10.3389/fped.2021.698577

**Published:** 2021-08-06

**Authors:** Karim N. Daou, Abir Barhoumi, Amina Bassyouni, Pascale E. Karam

**Affiliations:** ^1^Department of Pediatrics and Adolescent Medicine, American University of Beirut Medical Center, Beirut, Lebanon; ^2^Department of Nutrition, American University of Beirut Medical Center, Beirut, Lebanon; ^3^Inherited Metabolic Diseases Program, Department of Pediatrics and Adolescent Medicine, American University of Beirut Medical Center, Beirut, Lebanon

**Keywords:** tyrosinemia, outcome, nitisinone, developing countries, liver transplant

## Abstract

**Background:** Hereditary tyrosinemia type 1 is a rare genetic disorder leading to liver cirrhosis and hepatocellular carcinoma. Few decades ago, dietary measures and ultimately liver transplant constituted the only treatment modalities. Nowadays, early diagnosis and therapy with nitisinone can reverse the clinical picture. In developing countries, diagnostic and therapeutic challenges may affect the outcome of this disease. The choice of the treatment modality may depend on the economic status of each country. Few reports on the long-term outcome of hereditary tyrosinemia type 1 are available from developing and Arab countries.

**Methods:** A retrospective study of charts of Lebanese patients diagnosed with tyrosinemia type 1 and followed, at the American University of Beirut, during a 12-year period was performed. Clinical presentation and liver biochemical profile at diagnosis were analyzed, along with therapeutic modalities and long-term outcome.

**Results:** Twenty-two children were diagnosed and followed during the study period. Median age at diagnosis was 7 months (range: one day to 35 months). Most of the patients presented with hepatomegaly and jaundice. Four patients were referred for atypical presentations with developmental delay and seizures, secondary to undiagnosed hypoglycemia episodes. Around half of the patients presented with failure to thrive. Transaminitis, cholestasis and increased α-fetoprotein level were variably present at diagnosis (36% to 50%). All patients had elevated plasma tyrosine and urinary succinylacetone levels. Genetic testing was performed in 9%. Only one third could be treated with nitisinone. Liver transplant was electively performed in 9% of cases, to overcome the long-term cost of nitisinone. One third of the patients died between the age of 1 month and 11 years. Surviving patients are still candidates for liver transplant.

**Conclusion:** Our experience reflects the challenges of diagnosis and treatment of hereditary tyrosinemia type 1 in a developing country. In the absence of specific neonatal screening, early diagnosis relies mostly on the clinical awareness of the physician. Long-term nitisinone use may be deterred by its high cost and liver transplantation carries risks of surgical complications. New, effective, and less expensive treatments are needed, especially for developing countries.

## Introduction

Hereditary tyrosinemia type 1 (HT1) is an autosomal recessive inborn error of tyrosine metabolism caused by a deficiency of fumarylacetoacetate hydrolase leading to the accumulation of succinylacetone (SA) and its precursors, responsible for progressive hepatic, renal and neurological damage (1). Clinical presentation of HT1 can be heterogeneous, even within the same family, including acute, subacute or chronic liver disease associated to Fanconi renal tubular acidosis and hypophosphatemic rickets (2). Failure to thrive can be an additional presenting feature in infancy and in chronic forms of childhood (3). Cardiomyopathy, porphyria-like neurological crisis, abdominal pain and polyneuropathy (2) have also been reported in HT1 patients. Biochemical diagnosis may be suggested by a non- specific elevation of plasma tyrosine and methionine levels, disturbed liver function with high plasma α-fetoprotein level (4) and proximal renal tubulopathy. An elevated SA level in blood or urine is diagnostic of HT1 (1, 5). SA in blood can be detected by Tandem Mass Spectrometry with high specificity and sensitivity (6) thus, allowing HT1 diagnosis by neonatal screening. Further confirmation relies on genetic sequencing of fumarylacetoacetate hydrolase gene (1).

In countries where neonatal screening for SA is not available, HT1 diagnosis remains challenging since clinical presentation can be variable and non-specific. Almost half of the patients are estimated to die undiagnosed (1), and late-diagnosed patients may still be encountered. Moreover, in Arab countries with high consanguinity rates, this autosomal recessive disorder is expected to be common. Clinical suspicion relies on the physician's awareness of HT1 myriad of symptoms leading to diagnosis and allowing therapy initiation.

Therapeutic options include nitisinone along with a dietary restriction of phenylalanine and tyrosine, with few indications left for liver transplantation. Before the nitisinone era, made available in 1992 (7), tyrosinemia patients- presenting before 2 months of age with acute liver failure- had a 2-year survival rate of 29% (8). Liver transplantation at that time was the sole possible choice, especially for patients who develop hepatocellular carcinoma (HCC). Nowadays, liver transplantation is still indicated in some cases (8) with a long-term survival of 85% (9, 10) despite various possible post-operative complications (11). Although nitisinone treatment significantly improved the outcome of patients with HT1, the main challenge remains the cost of this orphan drug and its availability in developing countries.

Few reports on the outcome of HT1 from Arab countries are available, mainly from Egypt (12), Saudi Arabia (13), Tunisia (14), and Sudan (15). Furthermore, some developing countries, like Mexico (16, 17), still struggle to diagnose early HT1 patients and offer them nitisinone, an expensive lifelong medical therapy. The aim of this study is to report the diagnostic and therapeutic challenges of a series of HT1 patients and their outcome over a 12-year- period, in a tertiary care center in Lebanon.

## Methods

A retrospective review of charts of HT1 patients diagnosed and followed at the Inherited Metabolic Disease Program of the American University of Beirut Medical Center-Lebanon, the oldest and largest referral center in the country, from January 2007 to January 2019 was carried out. Collected data included: gender, consanguinity, presence of HT1 cases in the family or unexplained deaths with liver failure, age at onset and at diagnosis, and clinical presentation. In addition, z-score for growth parameters at presentation and last follow-up were reviewed and analyzed.

Diagnostic tests included liver function tests: aspartate transaminase, alanine transaminase, alkaline phosphatase, γ-glutamine transaminase, as well as prothrombin time, alpha-fetoprotein, SA and plasma aminoacids chromatography. SA levels were determined in blood by Tandem Mass Spectrometry or in urine by Gas Chromatography-Mass Spectrometry, depending on availability.

Treatment modalities, including diet, nitisinone and/or liver transplantation were reviewed. Target nitisinone range between 40 and 50 μmol/l was adopted to achieve normal SA levels.

For patients treated with nitisinone, serial blood levels of transaminases, plasma aminoacids (phenylalanine and tyrosine) were done weekly after initiation of therapy until normalization then monthly for the first 3 months, and every 3 months thereafter. All other patients were monitored every 3 months with same serial laboratory testing.

Follow-up duration, side effects of each therapeutic modality and outcome were also recorded.

Family screening was performed for all alive siblings by plasma aminoacid chromatography, and/or genetic testing, when available.

This study was approved by the Institutional Review Board of the American University of Beirut (protocol number: PED.PK.03 and BIO-2018-0381).

### Statistical Analysis

Median data and range for continuous variables, frequency and percentage for categorical variables were calculated. Data analysis was performed on SPSS software version 23.

## Results

Twenty-two children were diagnosed with HT1 and followed between January 2007 and January 2019 at the Inherited Metabolic Diseases Program of the American University of Beirut Medical Center-Lebanon (Table 1). Consanguinity was noted in 77% of families, with a male predominance among patients (73%). Half of the patients had a positive family history of unexplained siblings' death with undiagnosed liver disease in 41% of them.

**Table 1:** Clinical presentation, treatment, and outcome of Hereditary Tyrosinemia type 1 patients in Lebanon.

**Patient**	**Family history^**†**^**	**Age at onset**	**Age at diagnosis**	**Clinical presentation**	Height^*^	Weight^*^	**Treatment**	**Follow up duration**	Height^**^	Weight^**^	**Outcome**
1/M∧	No	3 d	3 m	HMG, jaundice, FTT	−3.56	−3.55	Diet	4 m	−3.33	−3.16	Alive
2/M∧	No	6 m	24 m	Delay, seizures, HMG, FTT	−2.85	−2, 75	Diet	11 y 2 m	−3.81	−3	Alive
3/F∧	Yes	3 m	5 m	HMG, jaundice, FTT	−3.91	−3.58	Diet	7 m	−3.81	−3	Alive
4/M∧	No	3 d	24 m	Delay, seizures, HMG, FTT	−2.65	−2.8	Diet	12 y	−3.65	−2.39	Alive
5/M∧	No	1 d	12 d/NBS	HMG, jaundice	−1.83	−0.65	Nitisinone	4 y	−1.56	0.45	Alive
6/F	Yes	3 m	6 m	Jaundice, HMG, SMG, ascites	−1.07	−1.53	Diet	10 y 3 m	1.45	1.75	Died (HCC)
7/M∧	Yes	14 m	24 m	HMG, SMG, jaundice, ascites, GI bleeding	−1.2	−0.46	Diet	11 y	0.25	−0.7	Died (HCC)
8/M∧	No	15 m	35 m	Delay, seizures, HMG	1.79	−0.56	Diet	3 y	1.68	0.46	Alive
9/M∧	Yes	2 m	2 m	HMG, jaundice	−1.23	−1.16	Diet	3 m	−1.2	−1.1	Alive
10/F∧	No	7 m	21 m	HMG, SMG, ascites, FTT, neuropathy	−2.87	−2.64	Diet	3 m	−2.85	−2.57	Alive
11/M∧	No	2 d	1 m/NBS	Jaundice	−1.25	−1.32	Diet, LT	2 m	−1.2	−1.29	Died (LF)
12/M∧	No	14 m	24 m	HMG, FTT	−5	−3	Nitisinone, LT	12 y	−4.32	−1.65	Alive
13/M	No	20 m	25 m	HMG, jaundice, SMG, epistaxis, ascites	0.2	0.52	Nitisinone	3 y 1 m	0.86	1.96	Alive
14/F∧	Yes	16 m	19 m	HMG, SMG, ascites	0.32	0.42	Nitisinone	2 y 3 m	−0.35	0.08	Alive
15/M∧	No	5 m	8 m	Delay, seizures, HMG	0.68	0.22	Diet	9 m	0.65	0.35	Alive
16/M	Yes	1 m	5 m	HMG, Jaundice, SMG, ascites	1.2	0.87	Diet	11 m	1	0.75	Alive
17/M∧	Yes	2 m	4 m	HMG, SMG, ascites, FTT	−3.5	−3.87	Diet	10 m	−3.2	−3.9	Died (LF)
18/M	No	1 d	20 d	HMG, jaundice, hypoglycemia	0.33	0.57	Diet	1 m	0.3	0.45	Died (LF)
19/M	No	1 d	19 d/NBS	Jaundice, SGA	−2.6	−3.65	Nitisinone	1 y	−0.73	−0.78	Alive
20/F∧	Yes	6 m	8 m	HMG, jaundice	−1.68	−1.5	Diet	7 y	−0.68	−1	Died (LF)
21/M∧	Yes	7 m	10 m	Jaundice, FTT	−2.4	−3	Nitisinone	2 y	−2.1	−2.6	Died (LF)
22/M∧	No	1 d	2 m	Jaundice, SGA	−3.1	−2.5	Nitisinone	4 m	−3.3	−2.7	Alive

*M, male; F, female; d, days; m, months; y, years; NBS, newborn screening; HMG, hepatomegaly; FTT, failure to thrive; SMG, splenomegaly; GI, gastrointestinal; SGA, small for gestational age; LF, liver failure; HCC, hepatocellular carcinoma; LT, liver transplant; ∧, history of consanguinity;*

† *undiagnosed liver disease;*

* *Z-score at diagnosis;*

** *Z-score at last follow-up*.

### Diagnostic Data at Presentation

The median age at onset of symptoms was 3 months (range: 1 day to 20 months), while the median age at diagnosis age was 7 months (range: 1 day to 35 months). Three patients only were detected by newborn screening using Tandem Mass Spectrometry, with elevated plasma tyrosine level. Most of the patients (Table 1) presented with hepatomegaly (82%) and jaundice (64%). One third (32%) suffered from splenomegaly and ascites, associated to upper gastrointestinal bleeding and epistaxis in two patients (patient 13 and patient 7). Failure to thrive at diagnosis was recorded in 36% with z-score calculation below 2 standard deviations for height and weight, in parallel with detected renal tubulopathy, while 9% were born small for gestational age. Atypical presentations were also noted: four patients (18%) referred for developmental delay, secondary to undiagnosed hypoglycemia episodes and seizures (patients 2, 4, 8, and 15), were discovered to have an underlying hepatomegaly. Only one patient (patient 10) had signs of peripheral neuropathy with hepatosplenomegaly and failure to thrive at diagnosis.

Initial investigations of liver function at presentation (Table 2) revealed an increase in liver enzymes associated to prolonged prothrombin time and increased plasma α-fetoprotein in half of the patients. Cholestasis with elevated blood gamma-glutamyl transferase and alkaline phosphatase levels was found in 36% while direct hyperbilirubinemia was detected in 46%. Subsequently, plasma aminoacid chromatography showed an elevated plasma tyrosine level in all HT1 patients at presentation.

**Table 2:** Biochemical liver profile in patients with hereditary tyrosinemia type 1: median levels at diagnosis and follow-up.

***Therapy***	**Diet alone (** ***n*** **=** **14)**			**Nitisinone (** ***n*** **=** **7) with diet**				**Liver transplant (** ***n*** **=** **2)**		
**Level normal range**	**Initial**	**1 m**	**Follow-up**	**Initial**	**1 m**	**6 m**	**Follow-up**	**Initial**	**1 m**	**Follow-up**
***Plasma phenylalanine**0–1 m:38–137 μmol/l>1 m: 31–75 μmol/lTarget: normal range*	62	37	43	74	53	56	69	93	33	62
***Plasma tyrosine**0–1 m:55–147 μmol/l>1 m:22–115 μmol/lTarget*<*400 μmol/l*	474	210	362	565	150	113	173	759	104	130
***AST** 0–50 IU/l*	72	63	69	73	33	29	44	80	71	49
***AFP** 1–85 U/ml*	13,161	9754	14,412	15,060	538	79	82	12,161	5,640	3,059
***Plasma SA** 0-1.16 umol/l*				1.70	0.3	ND	ND			
***Urinary SA**0–3 mmol/mol creatinine*								34		
***Urinary SA (qualitative)***	**+**	**+**	**+**	**+**				**+**	ND	ND

*AST, Aspartate transaminase, AFP: α-fetoprotein. SA: succinylacetone; n: number of patients, m: months, ND: not detectable*.

Elevated urinary SA was detected qualitatively in all patients while quantitative levels were obtained in 9% with a median SA level at diagnosis of 34 mmol/mol (normal range: 0–3 mmol/mol creatinine). Genetic testing was performed only for two patients, for parental counseling purposes.

Abdominal ultrasound was positive for hepatomegaly at presentation in 82%, of which 32% were associated to splenomegaly and ascites. Liver biopsy performed in 23% of cases showed microvesicular steatosis, associated to micronodular cirrhosis in 9%.

### Therapeutic Modalities and Outcome

Long-term follow-up (Table 1) was carried out for a variable period between 1 month and 12 years (median 18 months). Most of HT1 patients (68%) were kept solely on a restricted phenylalanine and tyrosine diet along with symptomatic treatment, with target plasma tyrosine level below 400 μmol/l and normal phenylalanine level. Only one third of the patients (seven patients) had access to nitisinone therapy, as they benefited from financial coverage by a third-party payer or were able to pay the medication cost out of pocket.

The mean dosage of nitisinone was 1.2 mg/kg/day (range 0.6–2 mg/kg/day), adjusted according to the plasma nitisinone target level and/or normalization of SA levels in blood or urine. Good compliance was observed in 83% of cases with normalization of liver function within an average of 2 weeks and α-fetoprotein within 6 months. Duration of nitisinone therapy varied between 4 and 37 months (Table 1), with no reported side effects. Plasma tyrosine level was well-controlled with mild dietary restriction. One patient diagnosed at 10 months of age (patient 21) was treated with nitisinone for 2 years; he died 1 month after stopping therapy, due to lack of financial coverage by the third-party payer.

Liver transplant was electively performed in two patients as they could secure financial coverage of the surgical procedure by a non-governmental organization. One patient suffered from liver failure and died 1 week after transplant (patient 11) while the other (patient 12) was successfully transplanted at 10 years of age, after 2 years of nitisinone therapy.

One third of the patients (seven patients) died between the age of 1 month and 11 years from severe liver failure (four patients), HCC (two patients) and severe gastrointestinal bleeding (one patient).

The remaining surviving patients suffer from liver cirrhosis and portal hypertension, documented by abdominal ultrasound in all patients and liver biopsy in 30 % of cases. They are still candidates for liver transplant.

## Discussion

The worldwide incidence of HT1 is 1:100 000–1:120 000 with clusters of cases in Scandinavia and Quebec province (18). In countries like Lebanon, where consanguineous marriages reach 36% (19), HT1 is expected to be relatively common. During the 12-year period of the study, 22 patients were diagnosed at a single referral center, in a small country. however, several cases might have been undiagnosed, and maybe died in a clinical picture of acute, severe liver failure. Few reports of the outcome and treatment of HT1 in developing countries are available, with a limited number of patients (Table 3).

**Table 3:** Outcome of hereditary tyrosinemia type 1 in developing countries.

**Country**	**References**	**Study years**	**Patients (*n*)**	Population*	**Follow-up** ** duration (years)**	**Nitisinone** ** (*n*)**	**Liver transplant** ** (*n*)**	**HCC (*n*)**	**Survival** ** (%)**
Turkey	Aktuglu-Zeybek et al. (20)	1993–2016	42	78 million	23	41	8	5	71
	Gokay et al. (21)	2008–2015	7		7	7	0	2	86
	Ozçay et al. (22)	2006	3			3	3	3	100
	Büyükpamukçu et al. (23)	2006	5			-	3	4	75
	Arikan et al. (24)	2006	6			-	5	5	50
India	Shah et al. (25)	2016	4	1.3 billion	3	4	1	1	100
	Shah (26)	2013	3		5	3	0	0	100
Brazil	Seda et al. (27)	1993–2012	16	199 million	20	-	16	12	100
Iran	Bahador et al. (1)	2006–2011	36	74 million	5	12	36	5	100
Mexico	Fernández-Lainez et al. (16)	1995–2012	16	126 million	18	1	3	2	75
Egypt	El-Karaksy et al. (12)	2006–2009	22	84 million	3	10	3	16	68
Lebanon	Current study	2007–2019	22	6.8 million	12	7	2	2	50

n, number of patients; HCC, Hepatocellular carcinoma; ^*^Estimated population number in the last year of study. https://datatopics.worldbank.org/world-development-indicators/.

Clinical presentation was similar to series from other developing countries (12) with hepatosplenomegaly and failure to thrive. Interestingly, 18% of HT1 patients were initially referred for developmental delay and seizures, as liver involvement and secondary hypoglycemia were missed by the treating physicians.

In developing countries, like Lebanon, the main diagnostic challenge of HT1 would be to focus metabolic investigations to reduce the cost, in the absence of a third- party payer in most cases. Diagnosis was suspected based on the combination of clinical presentation and unexplained liver and/or kidney insult, in addition to neurological symptoms in some cases. Confirmatory diagnosis was obtained by urinary SA elevation; genetic testing was limited to 9 % of cases, similar to another study from Saudi Arabia (13).

Untreated HT1 patients have poor long-term survival with occurrence of liver failure, HCC or respiratory failure secondary to neurological crisis (8). Outcome of HT1 is related to early diagnosis and therapy; untreated HT1 patients have dismal prognosis with early death before 2 years of age while some chronic patients may survive up to 12 years of age (28). Furthermore, detection of HT1 in the neonatal period is key for better prognosis. Delay in initiation of nitisinone therapy may lead to a rapid progression of liver failure and quality of life deterioration in HT1 patients, as reported in a series from Mexico (16). In Turkey, where HT1 patients had more access to nitisinone at earlier stages of the disease, higher survival rates were observed (21). In the current study, 82% of patients were diagnosed beyond the neonatal period. Subsequently, when nitisinone was available, age of initiation of therapy varied between 1 and 25 months. Response to nitisinone was satisfactory in all patients during the follow-up period (between 4 months and 12 years), with normalization of liver and kidney functions. Dietary measures could not prevent HT1 complications despite plasma tyrosine levels control, as expected. Furthermore, in patients presenting with failure to thrive and short stature, growth did not improve with dietary control alone, as reflected by Z-score for weight and height at presentation and at last follow-up. Overall survival rate was 62%. The rate is significantly higher than in pre-nitisinone reports (8) but comparable to the reported 6-year survival rate of HT1 in Mexico (60%) (16). With nitisinone therapy, higher rates of 85% and 100% are reported in the Turkish and French series, respectively (21, 29), transient interruption of the treatment due to lack of health insurance coverage is common in developing countries (17). Several studies have shown that nitisinone interruption can lead to higher mortality rate in HT1 by HCC (20), in addition to triggering neurological crisis (30), rapid deterioration (31, 32) and death.

In our series, the difficulty of maintaining nitisinone as a long-term therapy was impeded by the financial burden and not by patients' compliance. The only patient who stopped nitisinone therapy died of severe acute liver failure and gastrointestinal bleeding, occurring one month after therapy withdrawal.

In parallel, liver transplantation compared to nitisinone was shown to carry a much lower cost per person-year in a cost-consequence analysis study conducted in Canada, one of the most developed countries (33). However, as per guidelines, it should be considered only in patients with suspected HCC and liver nodules (34). Although this surgical procedure may carry higher mortality (27, 31), it may be the only lifesaving intervention for HT1 in developing or low resources countries, as it offers a less costly and more sustainable solution on the long run. In our series, two HT1 patients underwent liver transplant (9%) with good response in one of them while the other suffered from post-operative complications and died.

Although liver transplantation was privileged in some centers in Turkey to overcome the long-term cost of nitisinone therapy (22–24), recent reports from Turkey (20, 21). Brazil (27) and India (25) adopted nitisinone for most HT1 patients. In other developing countries like Mexico (16), management of HT1 patients still faces the same challenges as in Lebanon, in terms of lack of coverage for nitisinone and liver transplantation indications.

## Conclusion

Our experience reflects the challenges of early diagnosis and treatment of HT1 in a developing country. In the absence of neonatal screening for SA, a high index of suspicion should be triggered clinically by the presence of unexplained hepatomegaly and/or biochemical liver disturbance at any age, with or without failure to thrive or renal tubulopathy. Atypical presentation with psychomotor delay and seizures may also reveal an underlying HT1. The diagnosis and treatment of this disorder in developing countries is really challenging and many patients remain undiagnosed. Given that nitisinone has availability problems and liver transplantation carries risks of surgical complications, there is a need for new, effective, and less expensive treatments, especially for developing countries. Other therapeutic options are currently being explored for this disease, such as the use of molecular chaperones (35) and gene therapy (36).

## Data Availability Statement

The original contributions presented in the study are included in the article/supplementary material, further inquiries can be directed to the corresponding author.

## Ethics Statement

The studies involving human participants were reviewed and approved by Institutional Review Board (IRB)–American University of Beirut, Beirut-Lebanon. Written informed consent from the participants' legal guardian/next of kin was not required to participate in this study in accordance with the national legislation and the institutional requirements.

## Author Contributions

KD collected data and wrote the first draft of the manuscript. ABas contributed to data collection. ABar contributed to data analysis. PK contributed for study design, data analysis, writing, and review of the manuscript. All authors contributed to the article and approved the submitted version.

## Conflict of Interest

The authors declare that the research was conducted in the absence of any commercial or financial relationships that could be construed as a potential conflict of interest.

## Publisher's Note

All claims expressed in this article are solely those of the authors and do not necessarily represent those of their affiliated organizations, or those of the publisher, the editors and the reviewers. Any product that may be evaluated in this article, or claim that may be made by its manufacturer, is not guaranteed or endorsed by the publisher.
